# Delayed Manifestations of a Case of Blunt Chest Wall Trauma‐Induced Pseudoaneurysm of the Mitral‐Aortic Intervalvular Fibrosa: A Case Report and Literature Review

**DOI:** 10.1002/ccr3.72178

**Published:** 2026-04-20

**Authors:** Alisina Mirzaei, Armin Attar, Alireza Moaref, Ahmad Ali Amirghofran

**Affiliations:** ^1^ Department of Cardiovascular Medicine Shiraz University of Medical Sciences Shiraz Iran; ^2^ Student Research Committee Shiraz University of Medical Sciences Shiraz Iran; ^3^ Cardiac Surgery Department Shiraz University of Medical Sciences Shiraz Iran

**Keywords:** blunt trauma, interventricular fibrosa, long‐term, pseudoaneurysm

## Abstract

Pseudoaneurysm of the mitral‐aortic intervalvular fibrosa (P‐MAIVF) is a rare condition resulting from dehiscence of the mitral‐aortic intervalvular fibrosa (MAIVF), leading to the formation of a sac communicating with the left ventricular outflow tract (LVOT). P‐MAVIF is caused mainly by infective endocarditis (IE) or trauma during medical procedures. Only six cases of blunt chest wall trauma‐induced P‐MAIVF have been reported, and our case is the first to report its long‐term complications. A 32‐year‐old man is reported with a history of a motorcycle accident that was referred due to a to‐and‐fro murmur. The echocardiographic evaluation one year after the accident revealed P‐MAIVF, and the patient was advised to undergo an operation, but he refused. Three years later, he returned with New York Heart Association (NYHA) class III dyspnea associated with orthopnea. A new transesophageal echocardiogram (TEE) confirmed a large P‐MAIVF with a dilated, dysfunctional left ventricle (LV). At this time, the patient underwent surgical intervention, and six months after the operation, he became asymptomatic, and cardiac function returned to normal. Our case is the first to report a long‐term complication of a blunt chest wall trauma‐induced P‐MAIVF, and our findings once again confirm the fact that early surgery and repair are a wise and appropriate decision for such patients.

## Key Clinical Message

A pseudoaneurysm of the mitral‐aortic intervalvular fibrosa is a rare complication of deceleration trauma. Although this condition may be missed during the initial post‐traumatic phase, increased awareness of this potential complication can facilitate timely diagnosis and appropriate management. This case highlights the importance of early surgical intervention to prevent disease progression and achieve favorable outcomes, as demonstrated by restoration of cardiac function and resolution of symptoms following timely repair.

## 1 Introduction

Mitral‐aortic intervalvular fibrosa (MAIVF) is a fibrous tissue between the aortic valve noncoronary cusp, the adjacent left coronary cusp, and the anterior mitral leaflet [1]. The pericardium roofs it and is inferiorly enclosed with the left ventricular outflow tract (LVOT) [2]. This area is comparatively avascular. Accordingly, injuries from infection/trauma lead to various abnormalities, including pseudoaneurysms and abscesses [3]. When dehiscence occurs in this region, a sac is formed between the aortic and mitral valves. A pseudoaneurysm of the mitral‐aortic intervalvular fibrosa (P‐MAIVF) can be generated by communication between the sac and the LVOT [4]. P‐MAIVF can rarely be observed; however, mortality is possible. P‐MAIVF causes are improper endocarditis, aortic valve surgery, congenital reasons, and blunt chest injuries [5]. Among these causes, blunt chest trauma is extremely rare. Here, for the first time, we report the late complications of a case of blunt chest wall trauma‐induced P‐MAIVF.

## 2 Case History/Examination

A 32‐year‐old man was referred to our clinic one year after a motorcycle‐related chest wall injury because of a newly detected to‐and‐fro heart murmur that prompted more cardiovascular evaluations. He was asymptomatic, and physical examinations were normal except for the mentioned murmur.

## 3 Differential Diagnosis, Investigations, and Treatment

**FIGURE 1:**
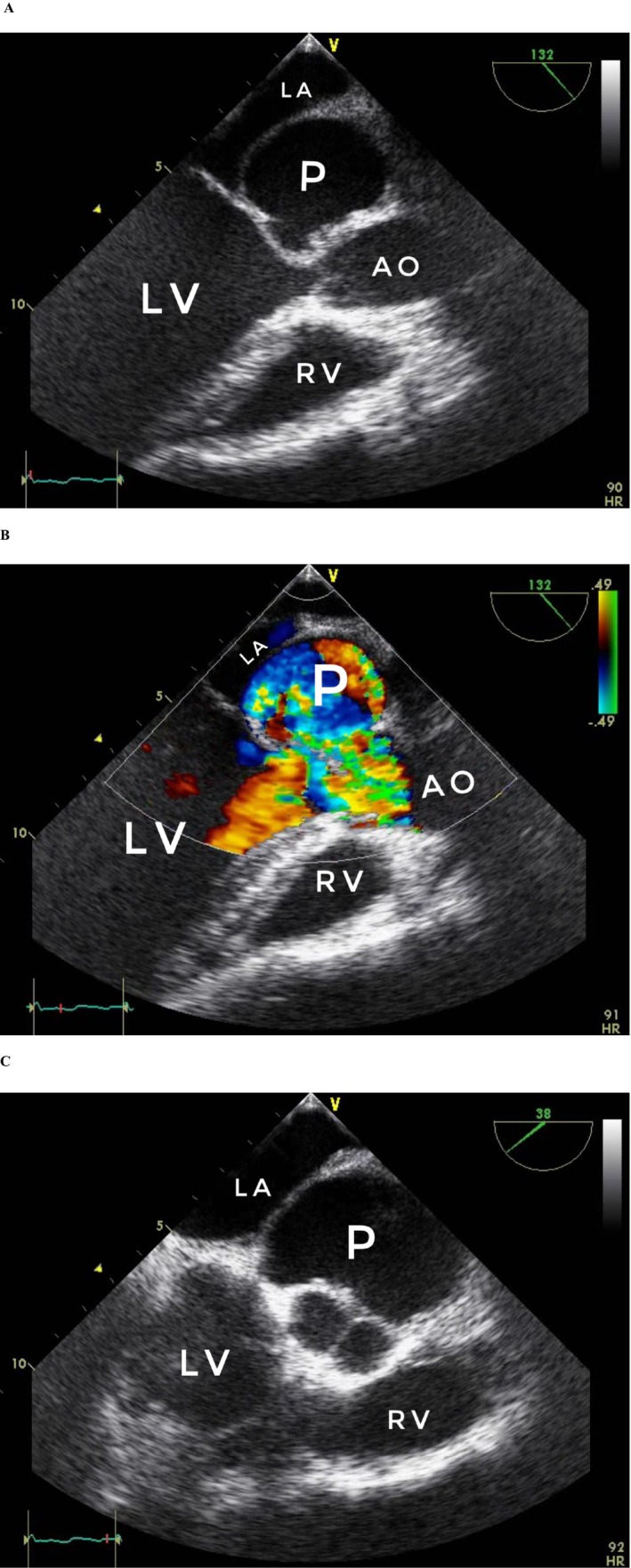
(A) Long axis view. (B) Long axis view. (C) Short axis view. Trans‐thoracic echocardiography short and long axis views. AO, aorta; LA, left atrium; L, left ventricle; P, pseudoaneurysm of the mitral‐aortic intervalvular fibrosa; RV, right ventricle.

Trans‐thoracic echocardiography (TTE) revealed a thin‐walled cavity in the area among the anterior mitral leaflet as well as the aortic root communicating to the LVOT with no connection to the left atrium (LA) or aortic root, which suggested P‐MAIVF (Figure 1). It is important to note that while the pseudoaneurysm was first detected on echocardiography one year after the trauma, this likely represents the time of initial detection rather than the lesion's formation. The pseudoaneurysm probably developed at the time of the initial blunt chest injury, but remained undetected for the first year due to the absence of follow‐up imaging studies during this period. The gradual enlargement and hemodynamic significance of the pseudoaneurysm over the subsequent years explains the delayed clinical manifestations and the worsening symptoms at the three‐year follow‐up.

No echocardiographic signs were found, indicating rheumatic heart disease or infective endocarditis (IE). The diagnosis was entirely based on echocardiography; unfortunately, no CT or MRI images are available. The patients reported no history of persistent fever, cough, or chest pain. His abdominal examination revealed no abnormal conditions. There were no skin lesions, and peripheral edema was found in the extremities. No acute rheumatic activity or IE was observed. The erythrocyte sedimentation rate was 35 mm/h. The C‐reactive protein concentration was 2.2 mg/dL, and the antistreptolysin O titer was normal. We advised him to undergo reparative surgery, but he refused. Three years later, he returned with NYHA class III dyspnea associated with orthopnea. A blood pressure of 100/60 mmHg and a heart rate of 112/min were recorded after his physical examination.

Following the lungs and heart evaluations, vast crackles, grade 4/6 pansystolic murmur at the apex, and gallops were observed. Electrocardiography revealed sinus tachycardia. A chest X‐ray showed cardiomegaly with a Cardiothoracic Ratio (CTR) of 0.55, which was suggestive of pulmonary edema. Routine blood evaluations revealed no abnormal range. TTE indicated a false aneurysm in the MAIVF at the LVOT, mild mitral regurgitation, and severe aortic regurgitation. The Left atrium (LA) and Left ventricle (LV) were dilated, and the ejection fraction decreased to 30% this time. This heart failure was caused by a severe shunt through the MAIVF fistula that formed, leading to hyperdynamic circulation that created the pseudoaneurysm.

## 4 Conclusion and Results (Outcome and Follow‐Up)

The patient was sent for an operation. The earlier diagnosis was reconfirmed during the operation, and a Bentall operation was performed. The reason for the Bentall operation was rupture of the aortic root and severe aortic insufficiency that required aortic root repair and aortic valve replacement. Six months after the operation, the patient's left ventricular systolic performance had recovered (LVEF: 52%), and he became asymptomatic again.

## 5 Discussion

P‐MAIVF, for the first time, was reported in 1966 by Waldhausen [6]. Infection, as well as surgical injuries in the MAIVF region, are among the most crucial reasons [7, 8]. Endocarditis may lead to abscess formation in the MAIVF area, and the resultant dehiscence in this region can lead to communication with the LVOT and the formation of a P‐MAIVF [8].

Endocarditis is the cause of P‐MAIVF in 63% of the reported patients in the literature; 93% of them had aortic valve endocarditis, and in the other 3%, the involved valve was the mitral valve alone [9]. In a case report, healed IE caused a P‐MAIVF rupture without either active endocarditis or a history of previous IE [10].

Patients with a bicuspid aortic valve may suffer congenital weakness in the MAIVF area; consequently, they are more prone to P‐MAIVF [11, 12]. Forty‐five percent of the patients with P‐MAIVF had a history of aortic valve surgery as the cause. Other cardiac surgeries or interventions, such as cardiac catheterization [13, 14, 15] Mitral valve surgery with the maze procedure [16] Radiofrequency catheter ablation for atrial fibrillation [2] The Ross/Konno procedure [13], and ventricular septal defect repair [17] may injure this region and lead to P‐MAIVF. Blunt chest trauma represents the rarest reported etiology.

To date, only six cases with a history of blunt chest injury have been reported [18, 19, 20, 21, 22], and all of them were operated on early. One was associated with coarctation of the aorta, a bicuspid aortic valve, and anomalous pulmonary venous return [18]. Therefore, this complication can be considered in patients with blunt chest trauma who also have valvular regurgitation.

Isolated clinical manifestations of this condition are uncommon, and most clinical presentations are manifestations of complications [8]. In a report by Sudhakar et al., 9% of the patients were asymptomatic, and among those who had symptoms, 39% presented with the clinical presentations of active endocarditis, 16% had signs and/or symptoms of heart failure, 12% had cerebrovascular accident and systemic embolism, and 10% presented with chest pain. The two most common complications were fistula generation and compression of the coronary arteries compression [8].

Since most cases of P‐MAIVF undergo early surgical correction to prevent rupture, the natural history of the disease is not fully clear. Pathophysiologically, they resemble valvular aortic regurgitation. The natural course for a few untreated patients has been reported, with a follow‐up of 10–63 months. For our patient, we performed a follow‐up of 63 months, and the pseudoaneurysm was the same size [13]. Another patient was followed up for four years, and the pseudoaneurysm size increased to between 1.0 cm and 1.7 cm [23]. Among those who died during follow‐up, 3 cases were caused by pseudoaneurysm rupture into the pericardium [12, 14, 24], three by heart failure [25, 26, 27], two by sepsis [28, 29], and one from a stroke [24]. Our case is the first to report a long‐term complication of blunt chest wall trauma‐induced P‐MAIVF, and our findings further support early surgical intervention as an appropriate management strategy for such patients.

## Author Contributions

**Alisina Mirzaei:** data curation, writing – original draft, writing – review and editing. **Armin Attar:** conceptualization, project administration, supervision, writing – original draft. **Alireza Moaref:** data curation, investigation, supervision. **Ahmad Ali Amirghofran:** data curation, investigation.

## Funding

The authors have nothing to report.

## Consent

The patient provided complete written informed consent for the publication of this study and its accompanying images, in accordance with the journal's patient consent policy.

## Conflicts of Interest

The authors declare no conflicts of interest.

## Data Availability

The data that support the findings of this study are available from the corresponding author upon reasonable request.
